# Supplementary material for: Morphological pseudotime ordering and fate mapping reveal diversification of cerebellar inhibitory interneurons
Source: Nat Commun. 2022 Jun 14;13:3433. doi: 10.1038/s41467-022-30977-2 (PMC9197879; doi:10.1038/s41467-022-30977-2)
Supplement: Supplementary file 1 — Supplementary Information [file 41467_2022_30977_MOESM1_ESM.pdf]

Supplementary Information

Morphological pseudotime ordering and fate mapping reveal diversification of cerebellar inhibitory interneurons

Wendy Xueyi Wang[1,2], Julie L. Lefebvre[1,2 #]

[1] Program for Neuroscience and Mental Health, Hospital for Sick Children,Toronto, ON Canada.
[2] Department of Molecular Genetics, University of Toronto.

[#] Corresponding author: julie.lefebvre@sickkids.ca

Supplementary Tables:

Supplementary Table 1: 27 morphological features used for morphometric and clustering analyses of mature MLI

Supplementary Table 2: 28 morphological features compiled for developmental pseudotime analyses.

Supplementary Figures:

Fig. S1: Overlapping distribution of morphometric parameters suggest that BC and SC m-types cannot be classified by a single feature

Fig. S2: SCs present significant within-class heterogeneity as confirmed through linear regression.

Fig. S3: MLI t-type marker expression in P7 cerebellum.

Fig. S4: Manual staging of MLIs into one of four maturation stages based on dendritic morphology.

Fig. S5: PHATE distribution of MLIs annotated by folia position, and removal of single parameters.

Fig. S6: Pvalb expression is upregulated in maturing MLIs in the lower ML, but not visible in immature migrating MLIs in the upper ML.

Fig. S7: Progressive growth and increased axonal measurements for MLIs during maturation.

Fig. S8: Palantir plots of early-born BCs annotated by manual maturity staging and cell age.

Fig. S9: Raw images for example reconstructions shown in Figure 9

Supplementary Table 1

**Dendritic Parameters**	**Axonal Parameters**	**Soma and Location parameters**
Dendritic length (µm)	Axon length (µm)	Folia*
Number of primary dendrites	Presence of axon carrying dendrite (0/1)	Molecular layer height*
Filopodia density (/100 µm)	Number of axon	Volume of somatic renders (μm^3)
Dendrite Z-depth (µm)	Axon Z-depth (µm)	
Height of ML covered (% ML)	Axonal span along ML (µm)	
Number of Sholl intersections at 10 µm	Height of ML covered (% ML)	
Number of Sholl intersections at 50 µm	Weighted number of baskets	
Number of Sholl intersections at 100 µm	Length of branches directed towards the apical ML*	
Number of Sholl intersections at 150 µm	Length of branches directed towards the basal ML*	
Length of branches directed towards the apical ML*	Percentage of branch length towards the apical ML	
Length of branches directed towards the basal ML*	Percentage of branch length towards the basal ML*	
Percentage of branch length towards the apical ML		
Percentage of branch length towards the basal ML*		

Supplementary Table 1. List of 27 morphological parameters compiled from 79 mature MLIs. Parameters denoted by an asterisk were removed during feature selection to avoid duplicated features. In total, 19 parameters were used for clustering in Fig. 2 and 3.

Supplementary Table 2

Axonal Parameters	Soma and Location Parameters	Dendritic Parameters
Axonal Length (µm)	Molecular layer height	Number of primary dendrites
Presence of axon carrying dendrite (0/1)	Soma diameter in X direction (µm)	
Maximum branch level	Soma diameter in Y direction (µm)	
Mean branch level	Soma diameter in Z direction (µm)	
Axon straightness (0 - 1)	Somatic volume (μm^3)	
Axonal span along ML (µm)	Folia*	
Axon Z-depth (µm)		
Number of pinceaux		
Number of full baskets		
Number of half baskets		
Number of branches contacting PC soma		
Number of branches directed towards apical ML		
Number of branches directed towards basal ML		
Length of branches directed towards apical ML (µm)		
Length of branches directed towards basal ML (µm)		
Number of Sholl intersections at 5 µm		
Number of Sholl intersections at 10 µm		
Number of Sholl intersections at 50 µm		
Number of Sholl intersections at 100 µm		
Number of Sholl intersections at 150 µm		
Number of Sholl intersections at 200 µm		

Supplementary Table 2. List of 28 morphological parameters compiled from 732 developing MLIs. Folia was removed from final pseudo-timeline generation to avoid bias, but used for projection of spatial information onto pseudotime (Supplementary Fig. 5a), and is denoted with an asterisk.

Supplementary Fig. 1

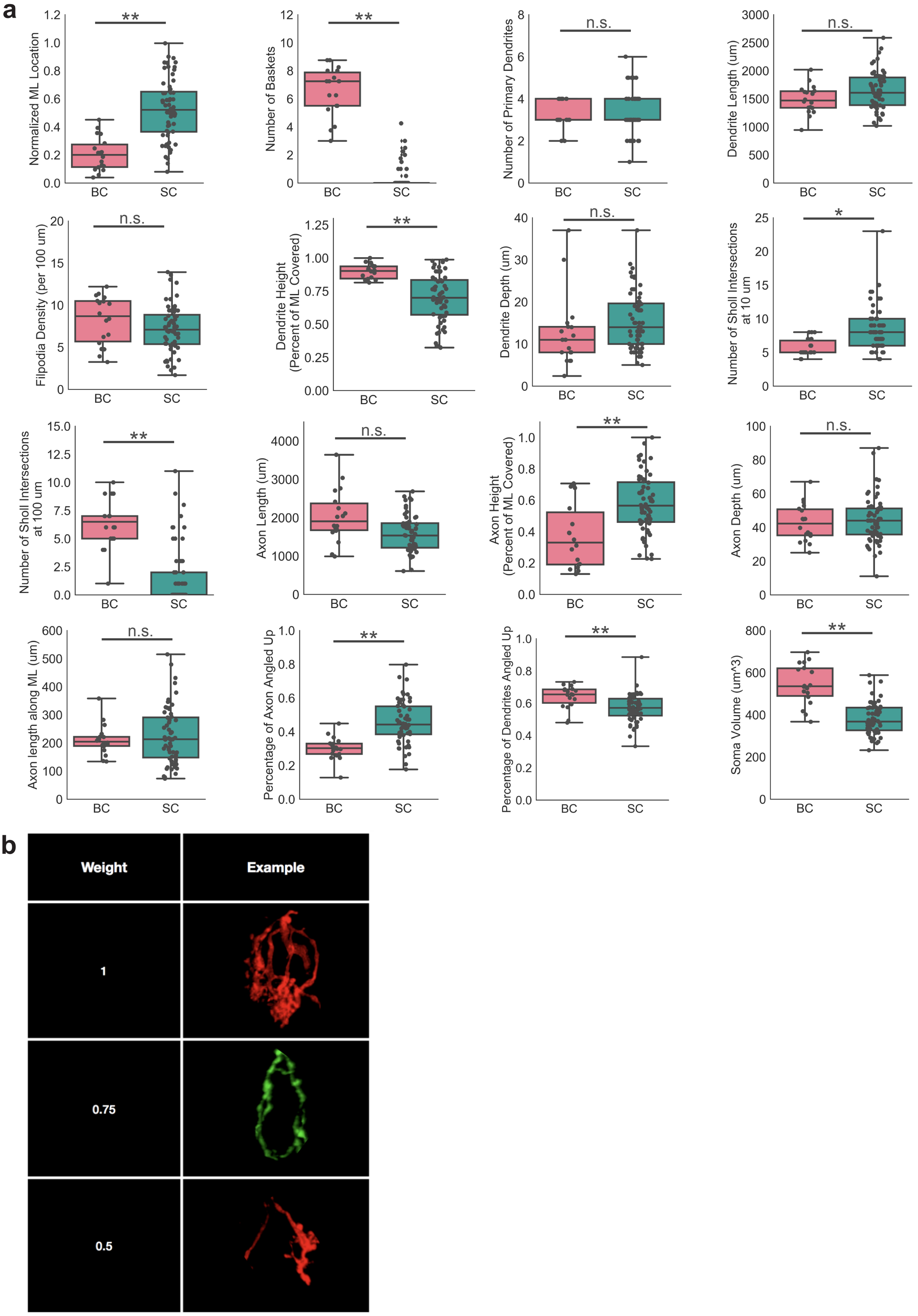

Supplementary Fig. 1 Overlapping distribution of morphometric parameters suggest that BC and SC m-types cannot be classified by a single feature. a Comparisons of morphometric parameters for the BC (pink) and SC (cyan) subpopulations divided by the clustering analysis in Figure 2 (n = 19 BCs; 60 SCs). Box plots indicate median (middle line); 25th, 75th percentile (box); and maximum and minimum (whiskers). Data points for all cells are superimposed onto the box plots. The magnitudes for each parameter overlap between BCs (pink) and SCs datasets (cyan), and thus no individual parameter is sufficient for MLI subtype identification. Statistical tests were performed using the non-parametric two-tailed Mann-Whitney's test. Asterisks denote significant differences; n.s, not significant. ML Location: p=0.000032; Number of baskets: p=0.00003; Dendrite height: p=0.000566; Number of Sholl Intersections at 10 µm: p=0.00165; Number of Sholl Intersections at 100 µm: p=0.0000413; Axon Height: p=0.0050; Percentage of Axon Angled Up: p=0.000115; Percentage of Dendrites Angled up: p=0.0152; Soma Volume: p=0.0000597. **b** Quantification scheme for PC-soma-targeting axon terminals (Weighted number of baskets, Supp. Table 1). Full baskets with pinceau are given weight of 1.0, full PC somatic innervation without pinceaux are given a weight of 0.75, somatic innervation which do not fully envelope the PC soma are given a weight of 0.5.

Supplementary Fig. 2

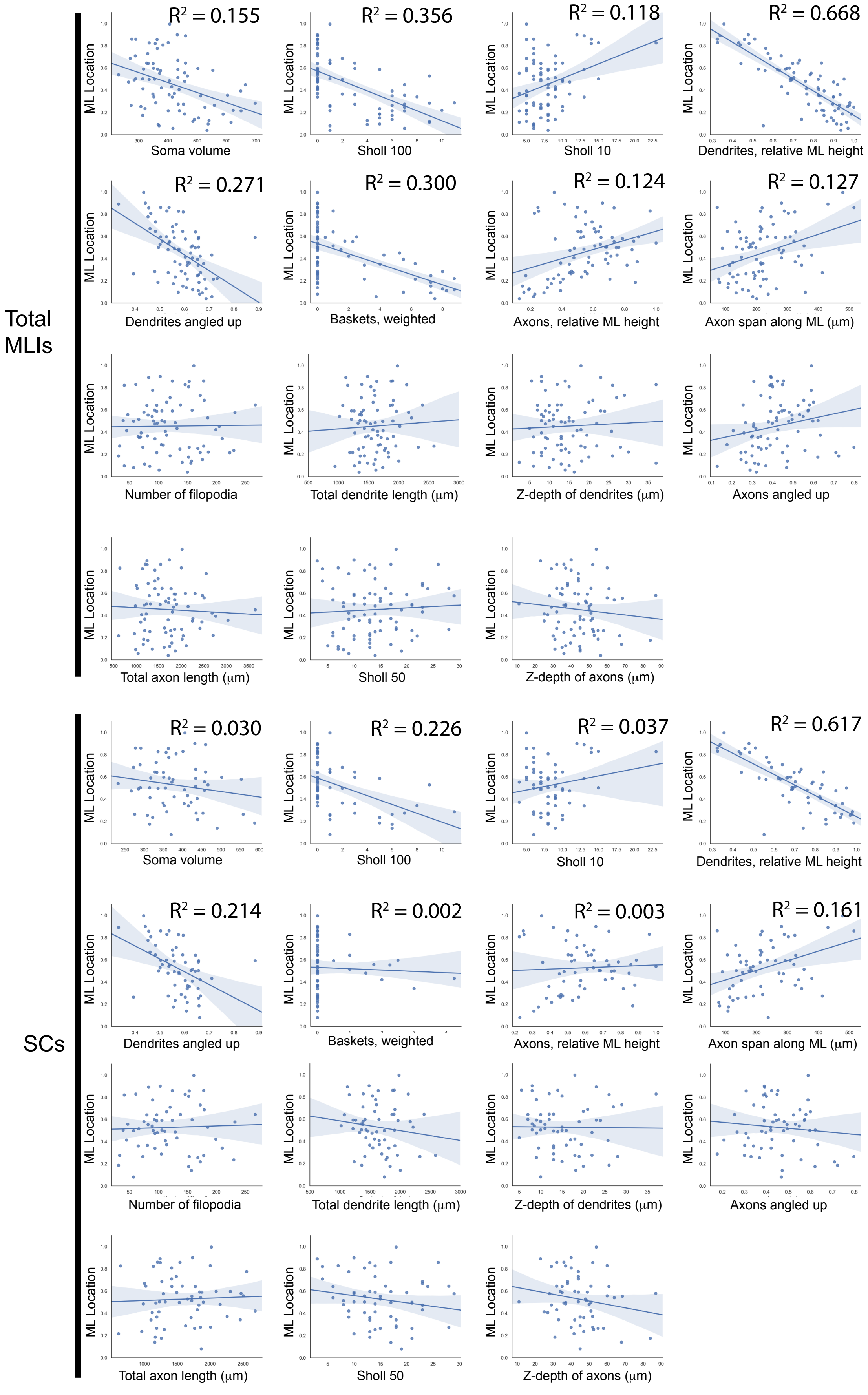

Supplementary Fig. 2 SCs present significant within-class heterogeneity as confirmed through linear regression. The top eight parameters show a positive or negative correlation to ML depth for the entire MLI population. Line represents linear model for regression, plotted with 95% confidence intervals.

Supplementary Fig. 3

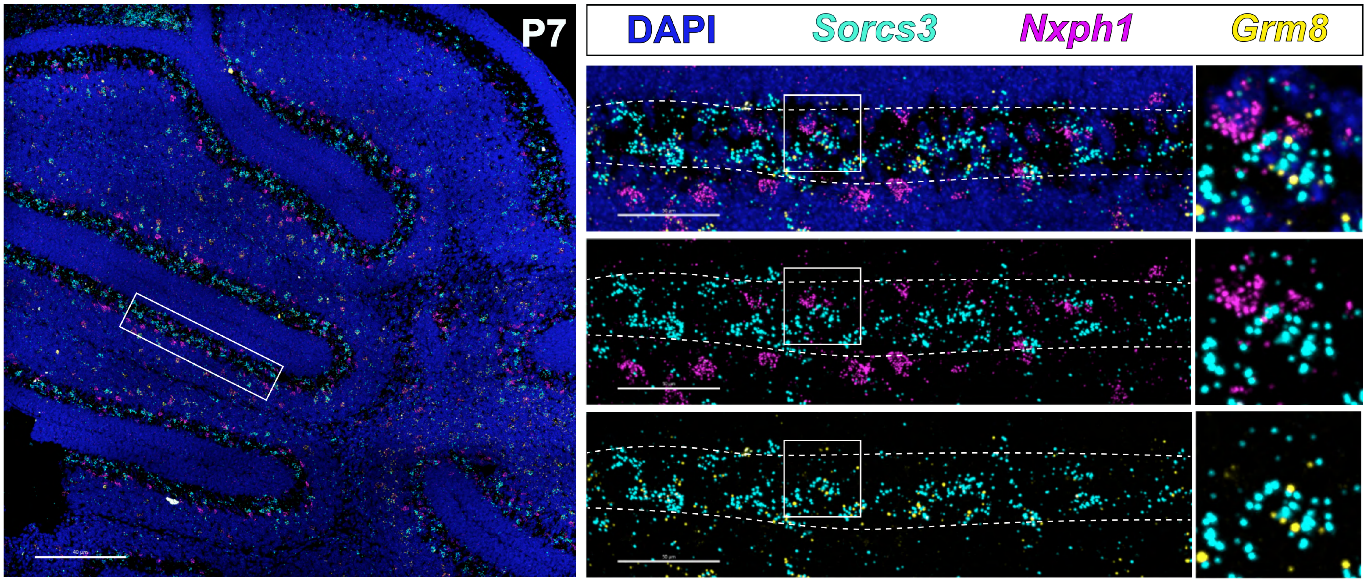

Supplementary Fig. 3 MLI t-type marker expression in P7 cerebellum. smFISH image of P7 cerebellum for *Sorcs3*, *Nxph1*, and *Grm8*. All three markers are observed within the ML. *Sorcs3* (cyan) and *Nxph1* (magenta) are expressed in largely non-overlapping cells, while *Grm8* expression (yellow) is observed at low levels in some *Sorcs3*+ cells. The right panel shows a zoomed in view of the ML, with the lower boundaries of two opposing ML outlined in white dashed lines. N = 2 animals. Scale bars are 40 μm (left) and 50 μm (right), respectively.

Supplementary Fig. 4

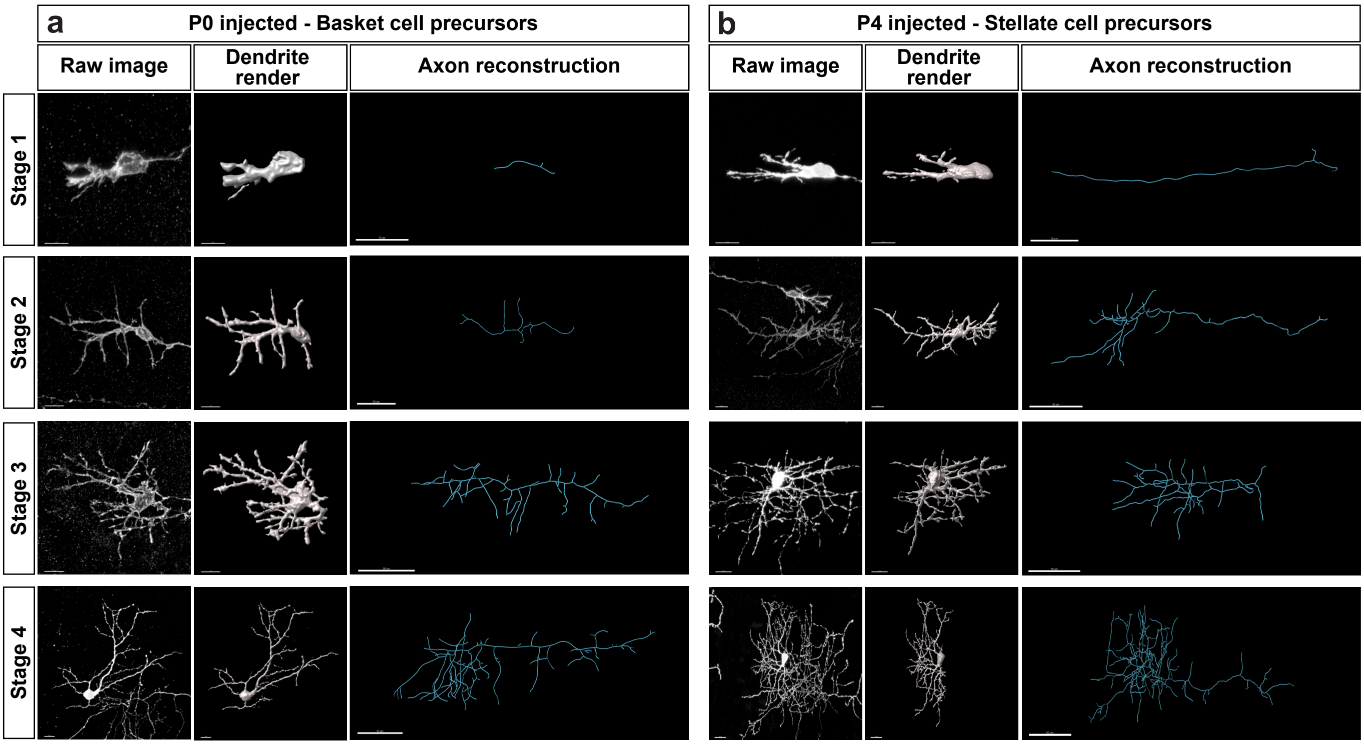

Supplementary Fig. 4 Manual staging of MLIs into one of four maturation stages based on dendritic morphology. a P0 injected presumptive BCs. **b** P4-7 injected presumptive SCs. Left, Representative images of presumptive BCs and SCs from the 732 developing MLI dataset. Cells were labeled by tamoxifen injection of Asc1-CreER; tdTomato or mTmG mice at P0 or P4, respectively. Middle, 3-dimensional rendering of the dendritic arbor. Each MLI was manually sorted into one of four maturation stages based on the extent of dendritic branching. Note the elongated soma orientated along the plane of dendrites in Stage 1 MLIs. Right, Complete reconstruction of the axonal arbor, which was measured by morphometric parameters listed in Supplementary Table 2. Manual staging of the developing MLI dataset was used to validate the PHATE- and Palantir-generated pseudotime ordering shown Fig. 8c and Supplementary Fig 8a, respectively. Scale bars are 10 μm in the left and middle columns, and 50 μm in the column on the right.

Supplementary Fig. 5

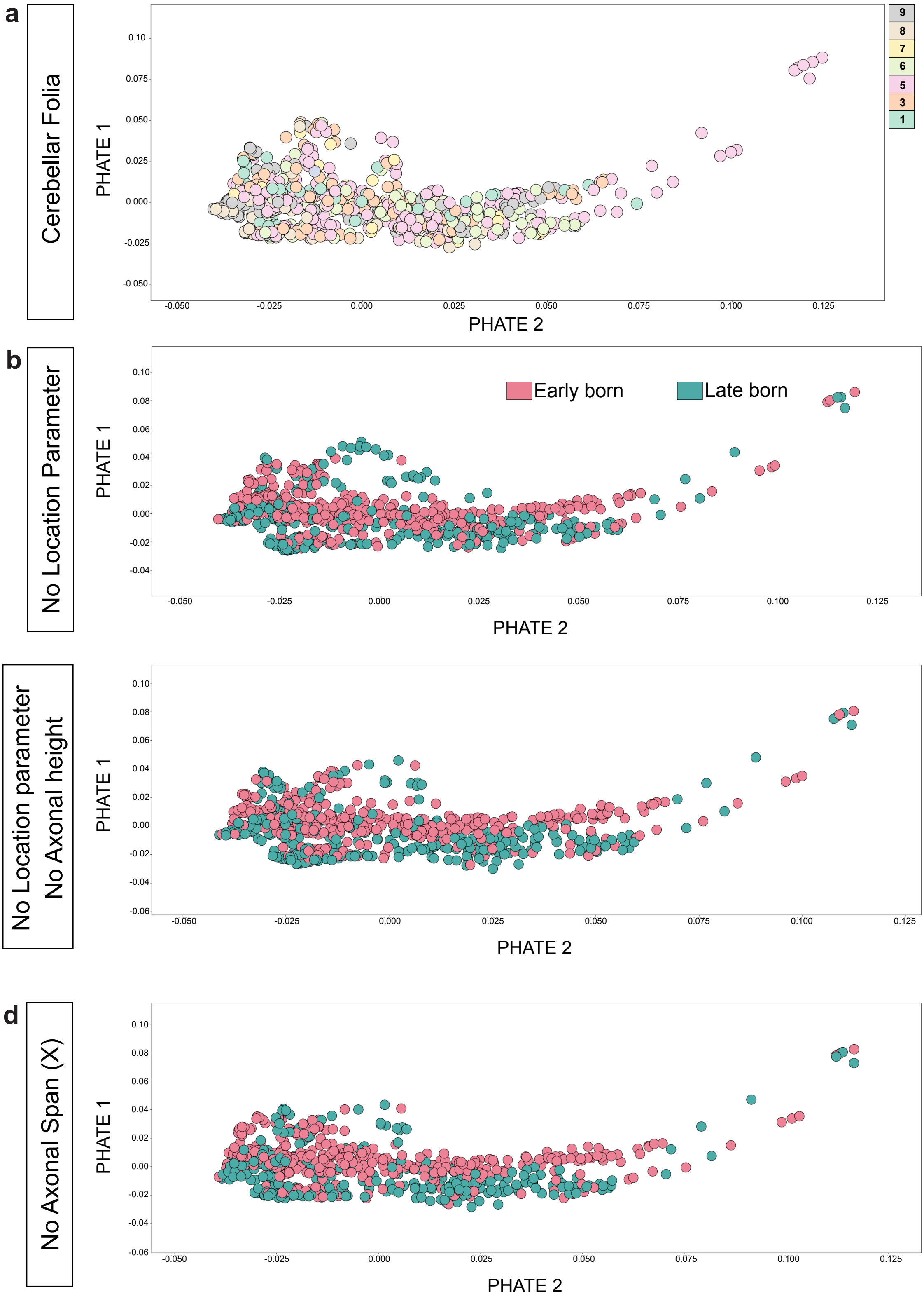

Supplementary Fig. 5 PHATE distribution of MLIs annotated by folia position, and removal of single parameters. a PHATE plot from Fig. 8b, with each developing MLI annotated by cerebellar folia location (folia 1-9). **b-d** PHATE statistics was re-calculated following removal of: **b** molecular layer position, **c** molecular layer position and axonal relative height coverage (vertical), **d** axon span (horizontal). Pink: Early born BC-fated population, P0 TMX-injected. Teal: Late-born SC-fated population, P4-7 TMX injected.

Supplementary Fig. 6

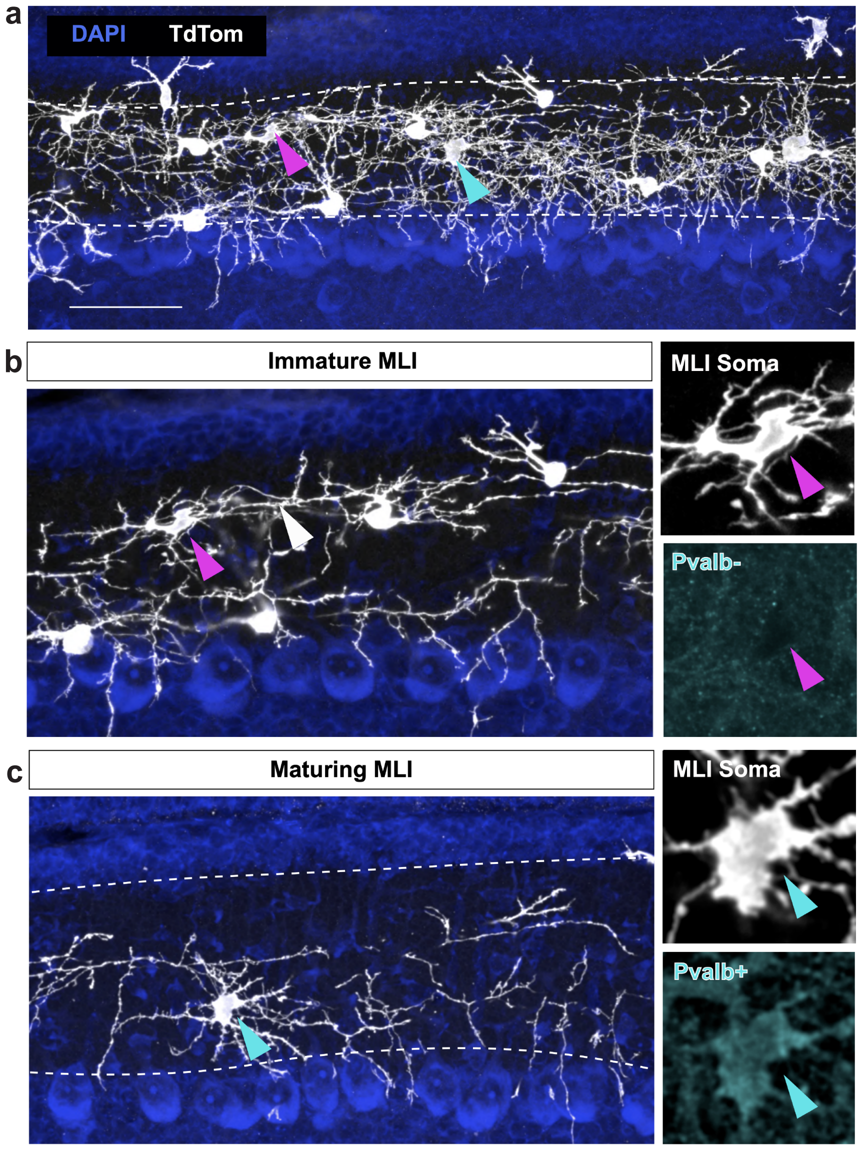

Supplementary Fig. 6 Pvalb expression is upregulated in maturing MLIs in the lower ML, but not visible in immature migrating MLIs in the upper ML. a Overview of nascent ML containing both migrating immature MLI precursors (upper ML; magenta arrow) and maturing MLIs (lower ML; cyan arrow). Boundaries of the ML are marked by the dashed lines. Scale bar is 50 µm. Sample is P0 TMX-injected early born MLI population from Ascl-CreER; TdTomato mice, captured at P10. **b** Co-immunolabeling for anti-Parvalbumin (Pvalb) with TdTomato-labeled MLIs (greyscale). Immature MLI example from **(a)**, marked by a magenta arrow, does not express maturation marker, Pvalb. The trailing process of this cell is labeled by the white arrow, a feature corresponding to cells in early stages of pseudotime. **c** Maturing MLI example from **(a)**, marked by a cyan arrow, expresses Pvalb (cyan). N = 3 animals.

Supplementary Fig. 7

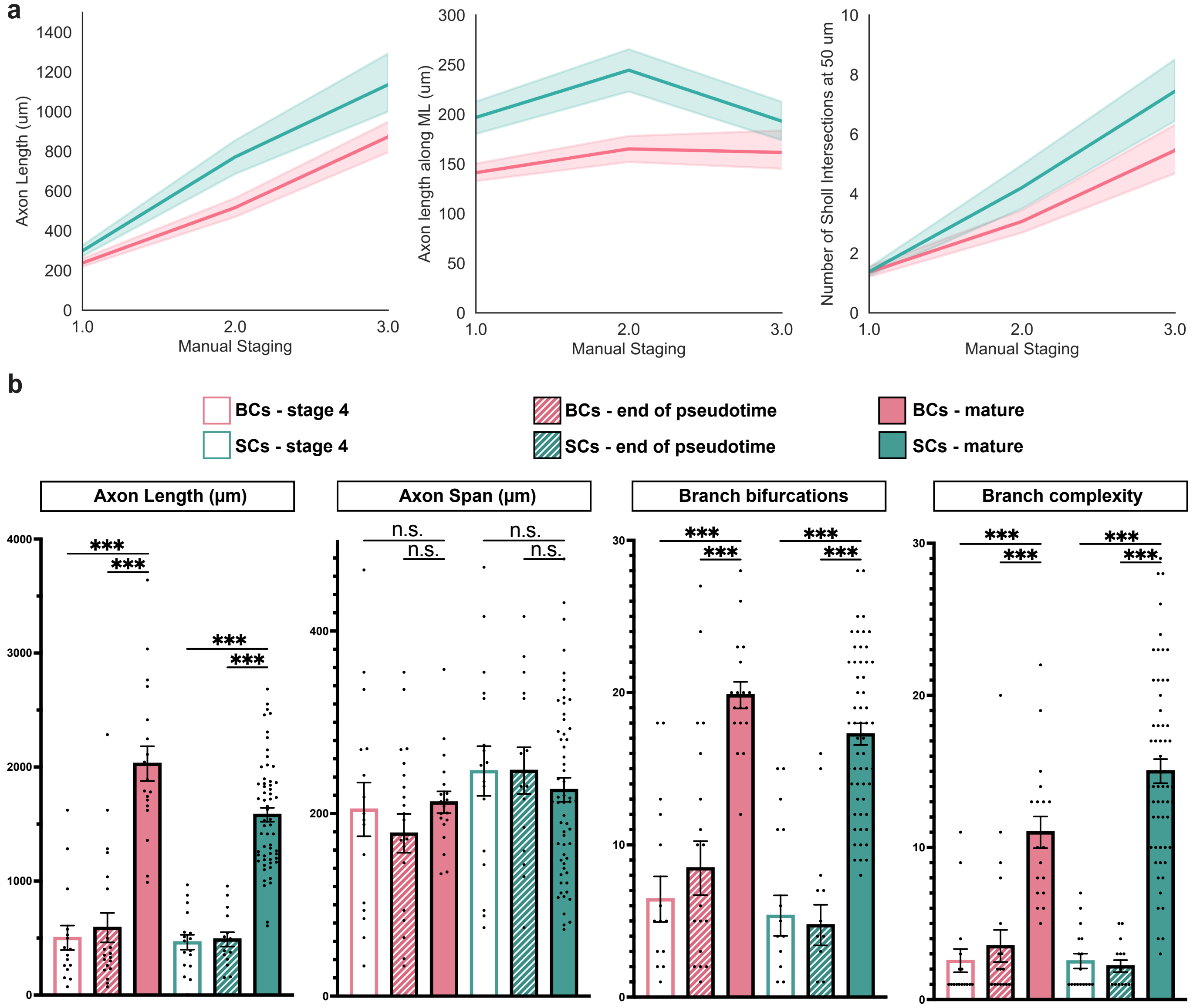

Supplementary Fig. 7. Progressive growth and increased axonal measurements for MLIs at final stages of pseudotime development and at maturity. a Line graph depicting progressive trends of individual morphological parameters over manual stages of maturation for the early-born presumptive BCs (P0-P1 TMX-injected, pink), and late-born presumptive SCs (P4 TMX-injected, teal). Solid lines show mean, and shaded bands show 95% confidence about the means. **b** Cells within stage 4 of manual staging (unfilled bars) or within the last 5% of pseudotime (semi-filled bars) were extracted from the respective BC and SC populations of the developing MLI dataset. BCs and SCs at the end of development were compared with their respective populations at maturity (P75, mature morphological dataset; filled bars) for axonal length, axonal span, the number of branch bifurcations from main axonal branch, and branch complexity as determined by Sholl analysis at 50 µm. Progressive growth was observed between the end of development and P75 for these parameters measured, except for axonal span for BCs and SCs. n= 19 mature BCs and 60 mature SCs. n = 16 stage 4 BCs and 17 stage 4 SCs. n = 21 BCs at end of pseudotime and 15 SCs at end of pseudotime. Bars show means and error bars represent SEM. Means were compared by two-tailed Mann Whitney test. Asterisks denote significant differences; n.s, not significant. Axon length (BC): p=0.0000015 stage 4 vs. mature; 0.0000019 end of pseudotime vs. mature. Axon length (SC): p=0.0000000008 stage 4 vs. mature; 0.000000009 end of pseudotime vs. mature. Axon span (BC): p=0.0000041 stage 4 vs. mature; 0.00005 end of pseudotime vs. mature. Axon span (SC): p=0.00000015 stage 4 vs. mature; 0.0000015 end of pseudotime vs. mature. Branch complexity (BC): p=0.0000075 stage 4 vs. mature; 0.000020 end of pseudotime vs. mature. Branch complexity (SC): p=0.0000000014 stage 4 vs. mature; 0.0000000053 end of pseudotime vs. mature.

Supplementary Fig. 8

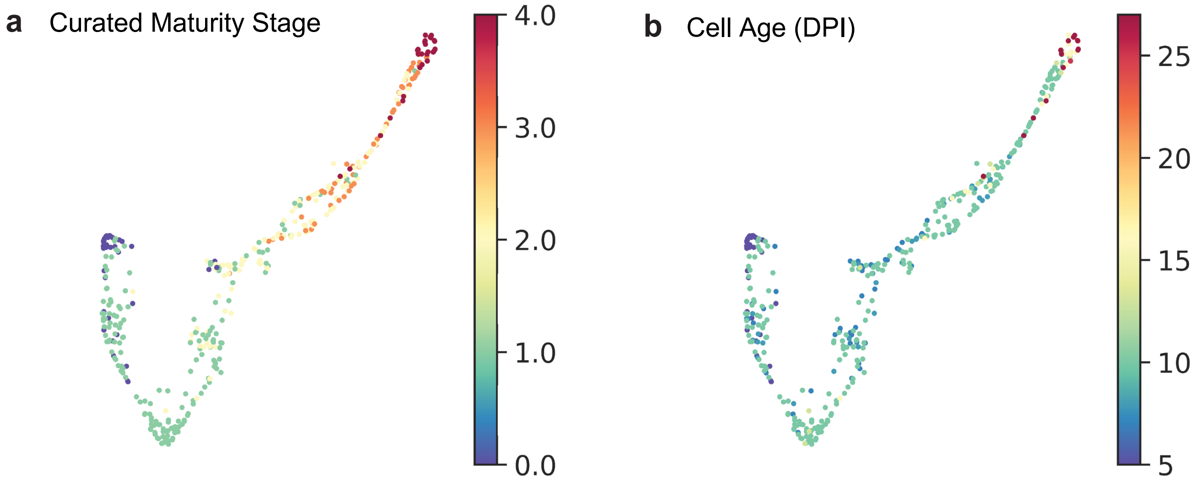

Supplementary Fig. 8 Palantir plots of early born BCs annotated by manual maturity staging and cell age. a Curated maturity stage for single MLIs projected onto Palantir trajectory. Stage 0 cells are MLI migratory precursors prior to axonogenesis. **b** Cell age projected onto Palantir trajectory as inferred from days after tamoxifen injection (DPI).

Supplementary Fig. 9

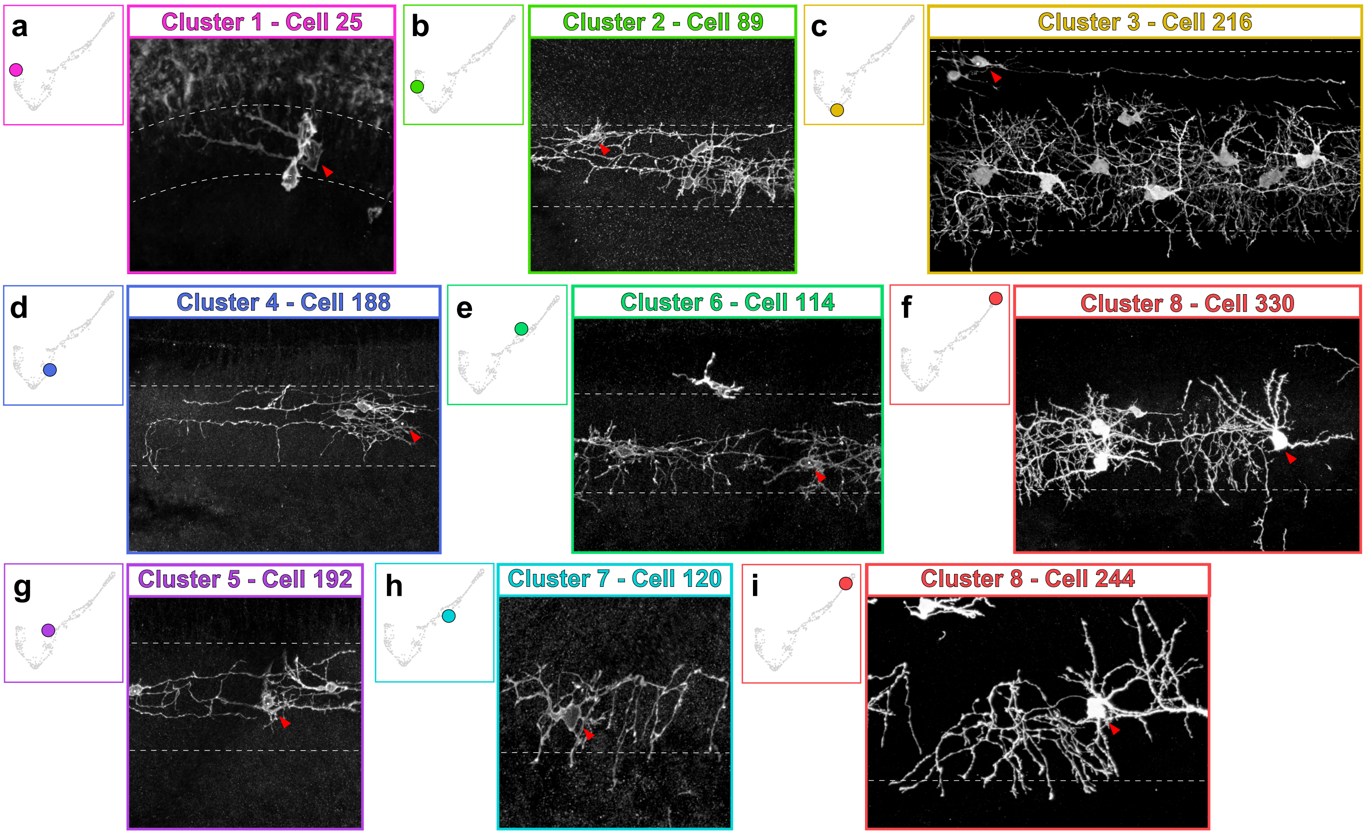

Supplementary Fig. 9. Raw images for example reconstructions shown in Fig. 9. Images represent raw confocal images used for each representative cell within Fig. 9. The somas for the cells of interest are marked by red arrows. Boundaries of the ML are marked by two dashed lines.
