# Supplementary material for: Morphological pseudotime ordering and fate mapping reveal diversification of cerebellar inhibitory interneurons
Source: Nat Commun. 2022 Jun 14;13:3433. doi: 10.1038/s41467-022-30977-2 (PMC9197879; doi:10.1038/s41467-022-30977-2)
Supplement: Supplementary file 3 — Reporting Summary [file 41467_2022_30977_MOESM3_ESM.pdf]

Corresponding author(s): Dr. Julie L. Lefebvre
Last updated by author(s): May 5, 2022

Reporting Summary

Nature Portfolio wishes to improve the reproducibility of the work that we publish. This form provides structure for consistency and transparency in reporting. For further information on Nature Portfolio policies, see our Editorial Policies and the Editorial Policy Checklist.

Statistics

For all statistical analyses, confirm that the following items are present in the figure legend, table legend, main text, or Methods section.

n/a	Confirmed	
☐	☒	The exact sample size (*n*) for each experimental group/condition, given as a discrete number and unit of measurement
☐	☒	A statement on whether measurements were taken from distinct samples or whether the same sample was measured repeatedly
☐	☒	The statistical test(s) used AND whether they are one- or two-sided<br>*Only common tests should be described solely by name; describe more complex techniques in the Methods section.*
☐	☒	A description of all covariates tested
☐	☒	A description of any assumptions or corrections, such as tests of normality and adjustment for multiple comparisons
☐	☒	A full description of the statistical parameters including central tendency (e.g. means) or other basic estimates (e.g. regression coefficient) AND variation (e.g. standard deviation) or associated estimates of uncertainty (e.g. confidence intervals)
☐	☒	For null hypothesis testing, the test statistic (e.g. *F*, *t*, *r*) with confidence intervals, effect sizes, degrees of freedom and *P* value noted<br>*Give P values as exact values whenever suitable.*
☒	☐	For Bayesian analysis, information on the choice of priors and Markov chain Monte Carlo settings
☐	☒	For hierarchical and complex designs, identification of the appropriate level for tests and full reporting of outcomes
☒	☐	Estimates of effect sizes (e.g. Cohen's *d*, Pearson's *r*), indicating how they were calculated

Our web collection on statistics for biologists contains articles on many of the points above.

Software and code

Policy information about availability of computer code

Data collection	LAS-X software (v. 2016 - 2021) was used to capture all images on a Leica SP8 confocal system.
Data analysis	Clustering and pseudotime analyses were performed in Python (v. 3.7.3) using Jupyter Notebook (v. 6.0.3). UMAP analyses were performed using the umap-learn (v. 0.3.9) and sklearn (v. 0.22.2) packages. Hierarchical clustering was performed using the ScipPy.cluster.hierarchy package. Palantir (v. 0.2.2; https://github.com/dpeerlab/Palantir) and PHATE (v. 1.0.2; https://github.com/ Krishnaswamylab/PHATE) trajectory inference were performed using published codes with a few modifications outlined in the Methods section. PAGA was performed using published code (v.1.6.0; https://github.com/theislab/paga). snRNA-Seq and smFISH clustering analyses were performed in Scanpy (v. 1.7.2) using published code (https://scanpy.readthedocs.io/en/stable/) and following parameters outlined in the methods section. Morphological reconstructions and feature extraction were performed in Bitplane Imaris (v. 8.2 - 9.5). Data visualization were performed using the Seaborn (v. 1.4.1) and Matplotlib (v 3.2.1) packages in Python, ggplot2 (v. 3.1.1) in R, or using Prism (v. 7.0c). XScope app for MLI counts was downloaded from the Apple App store (v. 3). Affinity designer (v.1.6.5) was downloaded from the affinity website. Code generated in this manuscript is available on Github located at https://github.com/wang-wendyy/Morphological_Pseudotime.

For manuscripts utilizing custom algorithms or software that are central to the research but not yet described in published literature, software must be made available to editors and reviewers. We strongly encourage code deposition in a community repository (e.g. GitHub). See the Nature Portfolio guidelines for submitting code & software for further information.

Data

Policy information about availability of data

All manuscripts must include a data availability statement. This statement should provide the following information, where applicable:

- Accession codes, unique identifiers, or web links for publicly available datasets
- A description of any restrictions on data availability
- For clinical datasets or third party data, please ensure that the statement adheres to our policy

Digital reconstructions generated in this study have been submitted to NeuroMorpho.Org under the Wang_Lefebvre archive [neuromorpho.org/WIN.jsp]. Morphometric data processed from the reconstructions and used for clustering (mature) and pseudotime trajectory inference (development) are provided in the Source Data file.

Field-specific reporting

Please select the one below that is the best fit for your research. If you are not sure, read the appropriate sections before making your selection.

☒ Life sciences ☐ Behavioural & social sciences ☐ Ecological, evolutionary & environmental sciences

For a reference copy of the document with all sections, see nature.com/documents/nr-reporting-summary-flat.pdf

Life sciences study design

All studies must disclose on these points even when the disclosure is negative.

Sample size	No statistical methods were used to calculate sample size as the level of heterogeneity was unclear prior to analyses. We sampled at comparatively exhaustive rates to similar studies. For mature analyses, we analyzed over three times more cells compared to a previous study of MLIs (Sultan and Bower, J Comp Neurol, 1998). Our sample size represents one of the largest morphological studies of any single neuronal population in the mouse brain (Gouwens et al., Nat Neurosci, 2019; Markram et al., Cell, 2015; Wang et al., Cell Rep, 2019; for example Que et al. Nature Comm. 2019 sampled 67 morphologies of Parvalbumin-expressing hippocampal interneurons). We did not calculate the sample size for our developmental dataset, which contains 732 cells across stages of cerebellar development from 32 individual mice and exceeds the numbers of cerebellar interneuron morphologies reported in previous studies (Telley et al. Neuron, 2016; Cameron et al. Dev. Biol. 2009; Ryan et al. Brain. Struct. Funct. 2017). We did not find studies that applied morphometric and multivariate analyses to mouse developing neurons at this scale.
Data exclusions	No data was excluded from the mature dataset. 5 (out of 732) radially migrating cells were excluded from the developmental dataset as they were outliers in pseudotime.
Replication	The mature analyses were performed across 9 mice (6 males, 3 females), with at least 4 cells per animal. Developmental analyses were performed across 32 mice from several litters (not sexed due to difficulties sexing pups; we did not genotype for sex). We did not observe animal-dependent biases in our analyses.
Randomization	Randomization was not applicable for data collection. All MLIs which were not cut off by the vibratome were imaged within stained tissue sections, and all cells were analyzed within images unless significant overlap with nearby cells rendered reconstructions difficult.<br><br>For classification of BC/SC m-type identities through subsampling, cells were randomly sampled using the random.sample() function in python.
Blinding	The experimenter was blinded to sample information, such as tamoxifen injection time point and animal age, while compiling and scoring morphological features. Clustering and pseudotime algorithms did not include experimenter-assigned annotations such as cell-type identity, maturity stage, times of tamoxifen injection, animal age, and cell age during analyses.

Reporting for specific materials, systems and methods

We require information from authors about some types of materials, experimental systems and methods used in many studies. Here, indicate whether each material, system or method listed is relevant to your study. If you are not sure if a list item applies to your research, read the appropriate section before selecting a response.

Materials & experimental systems

n/a	Involved in the study
	☒ Antibodies
☒	Eukaryotic cell lines
☒	Palaeontology and archaeology
	☒ Animals and other organisms
☒	Human research participants
☒	Clinical data
☒	Dual use research of concern

Methods

n/a	Involved in the study
☒	ChIP-seq
☒	Flow cytometry
☒	MRI-based neuroimaging

Antibodies

Antibodies used

Rabbit mCherry (cat#: EMU106), rat mTFP (cat#: EMU 104), guinea pig TagRFP (cat# EMU 108) from Kerafast; chicken GFP (cat #: GFP-1010) from Aves; rabbit RFP (cat# 600-401-379) from Rockland; goat Parvalbumin (cat# PVG-213) from Swant; mouse Calbindin (cat# C9848) from Sigma; and NeuroTrace 435/455 from Thermo Fisher Scientific. Donkey anti-goat AF647 (Life Tech, cat#: A21447); Donkey anti-goat AF488 (Life Tech, cat#: A11055); Donkey anti-rabbit AF488 (Jackson Immuno, cat#: 711-545-152); Donkey anti-rabbit AF568 (Life Tech, cat#: A10042); Donkey anti-rabbit AF647 (Jackson Immuno, cat#: 711-605-152); Donkey anti-chicken AF488 (Jackson Immuno, cat#: 703-545-155); Donkey anti-guinea pig AF647 (Jackson Immuno, cat#: 706-605-148); Donkey anti-rat AF647 (Jackson Immuno, cat#: 712-605-153).

Validation

All antibodies used are validated for IHC use by the manufacturer:

chicken GFP - analyzed by western blot and immunohistochemistry using transgenic mice expressing the GFP gene product.

Rabbit RFP - This product was prepared from monospecific antiserum by immunoaffinity chromatography using Red Fluorescent Protein (Discosoma) coupled to agarose beads followed by solid phase adsorption(s) to remove any unwanted reactivities. Assay by immunoelectrophoresis resulted in a single precipitin arc against anti-Rabbit Serum and purified and partially purified Red Fluorescent Protein (Discosoma). No reaction was observed against Human, Mouse or Rat serum proteins.

mouse Calbindin - The antibody does not react with other members of the EF-hand family, such as calbindin-D-9K, calretinin, myosin light chain, parvalbumin, S-100a, S-100b, S-100A2 (S100L) and S-100A6 (calcyclin). A weaker reactivity was observed with chicken calbindin-D-28K.

goat Parvalbumin - This antibody does not stain the brain of Parvalbumin knockout mice.

Brainbow mCherry, mTFP, and TagRFP antibodies were validated in a previous publication (Cai et al., Nat Methods, 2013). These antibodies were tested against a range of additional fluorescent proteins and no cross-reactivity were observed. We additionally validated all XFP antibodies in non-XFP expressing tissue.

Animals and other organisms

Policy information about studies involving animals; ARRIVE guidelines recommended for reporting animal research

Laboratory animals

All mice were maintained on a C57/B6J or mixed C57/B6J and FVB background. Strains used in this study include: Gad2-ires-Cre (Jax#: 010802); Ascl1-CreERT2 (Jax#: 012882); Ai14 Rosa-Cag-LSL-TdTomato (Jax#: 007908) and Rosa-mTmG (Jax#: 007579). Animals were P75 for the mature morphology dataset. Mice were P17 and P55 for the smFISH analyses in Fig. 6. Mice were P5, 7, 8, 10, 13, 14, 16, 25, and 27 for the developmental dataset. Animals of either sex were included in this study.

Mice were maintained on a 12 hour light/dark cycle, with lights on between 7AM and 7PM. Housing room temperatures were maintained between 21 - 23°C. Housing room humidity was maintained between 40-60%. Room lighting, temperature and humidity was controlled by the building automation system.

Wild animals

No wild animals were used in this study.

Field-collected samples

No field-collected samples were used in this study.

Ethics oversight

All experiments were carried out in accordance with the Canadian Council on Animal Care guidelines for use of animal in research and laboratory animal care under protocols approved by the Centre for Phenogenomics Animal Care Committee (Toronto, Canada) and the Laboratory Animal Services Animal Care Committee at the Hospital for Sick Children Research Institute (Toronto, Canada).

Note that full information on the approval of the study protocol must also be provided in the manuscript.
